# Comparing the effectiveness of Cognitive Behavioral Therapy and Stages of Change Model on Improving Abstinence Self-Efficacy in Iranian Substance Dependent Adolescents

**Published:** 2012

**Authors:** Mostafa Jafari, Shahriar Shahidi, Alireza Abedin

**Affiliations:** 1 Department of Psychology, Shahid Beheshti University, Tehran, Iran.

**Keywords:** Cognitive Behavioral Therapy, Self-Efficacy, Stages of Change Model, Substance Dependence.

## Abstract

**Objective:** The present study was conducted to compare the effectiveness of two therapeutic approaches, namely, Cognitive Behavioral Therapy (CBT) and Stages of Change Model (SOC) on improving abstinence self-efficacy in adolescent addicts.

**Methods:** Forty five self-referred adolescent addicts were randomly selected to take part in this study. Initial assessment was made using the following questionnaires: The University of Rhodes Island Change Assessment (URICA), General Self–Efficacy Questionnaire (GSE), and Adolescent Self–Efficacy Scale (ASES). Subjects were placed in two experimental (CBT, SOC) groups and one control group (three groups in all). The two experimental groups received twice a week interventions for 12 weeks and then were post tested and once again reassessed in a two-month follow up.

**Results:** Results clearly highlighted the effectiveness of the two models of intervention on general and special self-efficacy. The effectiveness of SOC proved greater than CBT on general self-efficacy in both posttest as well as the two-month follow up. Whereas CBT was more effective than SOC on special self-efficacy in posttest, SOC was more effective than CBT on dimensions of special self-efficacy in the follow up assessment.

**Conclusions:** Both CBT and SOC improve general and situational self-efficacy. Hence SOC may have more permanent and long lasting effect on self-efficacy than CBT.

## Introduction

Substance abuse in recent years has been considered as a social problem in Iran. 46 percent of drug addicts in Iran report that they began abusing drugs between the ages of 17 to 22 years (1). Hence, finding new ways of dealing with this problem has been one of the main priorities of the government in Iran (2). Research on self-efficacy and more specifically its application in therapeutic programs for drug users lately has attracted a good deal of attention and emphasis (3).

The term Self-efficacy based on Bandura's (4) Social-Cognitive Theory (SCT) indicates one's belief about how well change may occur successfully. Self-efficacy points to personal competency and sufficiency of one’s capability in executing tasks. The sources that produce these abilities include direct and vicarious experiences, verbal persuasion and physiological states (4).

SCT is interested in studying individuals’ direct experiences in controlling the environment toward effectively implementing behavioral changes. These experiences create a strong sense of efficacy (4) assumed useful for developing effective coping skills in a wide range of contexts such as relapse prevention in substance abusers (5).

Self-efficacy (6) is widely supported as a very important predictive factor in substance related problems (7). In alcohol studies, it is mainly considered as the level of confidence for coping with high-risk situations; it is the most important factor in predicting smoke cessation (8) and could predict 47 and 69 percent of the variance associated with alcohol and marijuana abuse respectively (9). Self-efficacy may be considered as an important yardstick in evaluating programs aimed at treating substance use disorders.

A popular form of intervention, which may be used in the treatment of addicts, is SOC. Used in a large number of studies investigating the treatment and resolution of substance use disorders, SOC is a frame work for understanding intentional behavioral change (10). This model is based on the assumption that behavior change takes place over time, passing through consecutive stages which are labeled as follows: pre-contemplation, contemplation, preparation, action and maintenance. This process is called natural consecutive steps toward permanent behavioral change (11). Addictive behavior in SOC model is viewed as a behavior that will respond to diverse forms of treatments at various stages of change. In this model levels and processes of change in addictive behavior is investigated in terms of attitudes, motivations, cognitions, and behaviors (12). In the change stage approach, self–efficacy and decision balance are two constructs that are among the main effective factors in initiating and maintaining substance use cession. Self-efficacy is a key factor in successful change. In SOC model, self-efficacy is estimated on the extent of the client's craving for the addictive substance and the extent to which he or she has self-confidence in refusing to engage in so called addictive behaviors (e.g. refusing to engage in the face of temptation) (12). Prochaska and Diclemente (11) believe that in the framework of SOC, in order to enhance the client's motivation to cease drug abuse, interventions based on the motivational change stages must be designed.

Hyde et al. (2008) in a Meta - analytical study, reviewed intervention programs in ten research projects aimed at improving self-efficacy in addicts. Results of their analysis showed that seven out of ten investigations reported significantly positive effects on patients' self-efficacy. It was concluded that self-efficacy can be improved by using different treatment methods (3).

The usefulness of SOC model has been confirmed as the treatment of choice with alcoholics in general (MATCH) (13), as part of alcohol prevention program for adolescents (14), in treatment of tobacco abuse, and in prevention of AIDS (12).

Likewise, cognitive behavioral therapy (CBT) has been reported to be effective in treatment of substance dependent patients (15). When compared with a variety of other treatments for alcoholism (16), CBT and coping skills training have ranked as either the best choice (17) or the second best (18).CBT’s effectiveness has been reported acceptable with adolescent substance abusers (19).

CBT distinctively provides appropriate methods for working with adolescents. The action-oriented methods are the most effective for adolescents along with the methods that help enhance adolescents’ sense of urgency for change in behaviors and thus improving motivation for treatment (20).

In recent years, the variety of studies on addiction psychotherapy has been vast. For example, in appraising the effectiveness of three methods, by matching different therapeutic approaches with specific individual problems, MATCH project showed that there were differences in the effectiveness of treatments; some therapies were more effective than others for some individuals.

Convergently, the aim of the present study was to compare the relative effectiveness of CBT and SOC on enhancing self-efficacy in Iranian adolescent addicts to abstain from drugs. It was hypothesized that CBT and SOC would both be effective in improving general and situational self-efficacy. Furthermore, the present study intended to investigate whether there would be a significant difference between the effectiveness of the two methods on general and situational self-efficacy. 

## Materials and Methods

*Subjects*

Forty five male adolescent volunteers referred to Greater Tehran Welfare Organization for treatment of substance abuse served as the subjects of the study. Their average age was 18.4 years (age range = 15 – 19) and the average duration of their drug use was 28 months.

*Design and procedure:*

A design with two experimental and one control group was used. Pre and posttests as well as a two-month follow up were implemented in the summer of 2008. Subjects were first screened using the Stages of Change Assessment (URICA, see below) and then randomly assigned to one of three groups. One group received SOC, another CBT and the third group received no treatment and was placed on a waiting list.

The procedures followed were in accord with the standards of the ethics Committee of department of psychology of Shahid Beheshti University. Reference number of approved study proposal is D/760/1066 on 2007-11-20.

*Instruments*

*University of Rhodes Island Change Assessment (URICA):*

This is a 32-item self-report measure that includes 4 subscales measuring the following stages of change: Pre contemplation, Contemplation, Action, and Maintenance. Responses are given on a 5-point Likert scale ranging from 1 (strong disagreement) to 5 (strong agreement). The subscales can be combined arithmetically (C + A + M – PC) to yield a second-order continuous Readiness to Change score that can be used to assess readiness to change at entrance to treatment (21).In a study conducted on 326 alcoholic out patients, (22) reported internal reliability of the scale and its subscales in the form of Cronbach's alpha to range from 0.77 to 0.79. High inter rater reliability of items by Iranian addiction experts as well as an overall alpha of 0.82 has also been reported (23).Test retest reliability after one month was reported to be 0.38, 0.49, 0.57 and 0.67 for pre contemplation, contemplation, action and maintenance respectively.

*General Self-efficacy Scale (GSES):*

This scale was designed to assess general self-efficacy (24). Split half reliability has been reported to be 0.76 for the overall score and in Iran reliability of 0.84 has been reported for this scale. Cronbach’s alpha was 0.85(25).

*Adolescent self-Efficacy Scale (ASES)*(26):

This scale was developed to assess situational self-efficacy in adolescents. According to Bandura (1977), self-efficacy percepts, that is the perceived ability to persevere in the continuing presence of stress, is an important factor in the individual’s assessment of his or her experience of mastery attained by effective performance. Furthermore, this perceived self-efficacy can and does vary from situation to situation (27). The properties of the scale were evaluated using a sample of 373 young 16 to 30 year old multiple drug users referred for treatment. Cronbach's alpha of this scale was reported 0.91. Construct validity, evaluated on a subset of the sample, was evident in significant correlations with concurrent measures of drug use severity and differential rates of changes in self-efficacy associated with two types of treatment. The ASES appears to be a reliable and valid scale for the measurement of self-efficacy in multiple-drug users.

*Pilot study*

A pilot study was carried out using 50 male undergraduate students at Shahid Beheshti University (mean age = 19.6, age range 18 – 24) prior to administration of the instruments on the subjects. This was done to ensure that the questionnaires were suitable for use in Iranian population. As a first step, the two questionnaires were presented to ten Iranian clinical psychologists working in the field of addiction in Tehran. These experts were asked to rate the suitability of the questions. Inter rater reliability for URICA was found to be 0.76 and for DASES was 0.54. The URICA and ASES were administered twice to subject with an interval of three weeks. Test retest reliability was thus measured and Pearson Correlation Coefficients of 0.38, 0.49, 0.57 and 0.68 were found for pre-contemplation, contemplation, action and maintenance stages respectively. For ASES, r was found to be 0.52, 0.61 and 0.49 for emotional, social and grief situations respectively. Cronbach's alpha by Splitting method also showed acceptable reliability for the questionnaires. The following alpha values were obtained: for overall URICA, an alpha of 0.82 and for overall ASES, an alpha of 0.93 was obtained.

## Results

As a first step, results on the URICA scale were analyzed and all subjects who were assessed to be at the “Action” stage of change were included in the main analysis. This meant that 45 out of 57 subjects tested were included. Table 1 shows means for the three groups at pre and posttest and two month follow up for general self-efficacy measure. 

Table 2 shows means and standard deviations for the three groups at pre and posttest and two month follow up for situational self-efficacy.

Multivariate Analysis of Covariance was conducted on the above data using the SPSS version 16 package. As can be seen from1, there is a difference between the two experimental groups (SOC and CBT) and the control group in general self-efficacy. For pre versus posttest, F = 30.72; df = 2, 43; p<0.01 and for posttest versus follow up, F = 23.74; df = 2, 43; p<0.01. This means that both CBT and SOC had a significantly more effect on general self-efficacy at posttest and follow up compared to the control group. As far situational self-efficacy is concerned and as can be seen from table 2, there is a significant difference between our two experimental groups and the control group. This was the case for all three self-efficacy situations (emotional, social and grief) at pre versus posttest and posttest versus follow up (Table 3).

**Table 1:** Descriptive data for general self-efficacy in three groups

**Depended variable**	**Between subjects**	**Between groups**	**Mean (standards deviation)**
General self-efficacy	pretest	CBT	54.06 (2.43) n=15
		SOC	54.86 (4.61) n=15
		Control	55.26 (5.17) n=15
	posttest	CBT	61.92 (3.30) n=13
		SOC	68.33 (3.79) n=12
		Control	56.57 (5.34) n=14
	follow up	CBT	65.53 (5.48) n=13
		SOC	76.25 (5.34) n=12
		Control	58.25 (6.15) n=12

**Table 2:** Descriptive data for situational self-efficacy in three groups

**Self-efficacy measures **	**le**	**Assessment **	**Conditions **	
				Mean (standard deviation)
Emotional situations		Pretest	CBT	
				46.40 (4.40) n=15
			SOC	45.66 (5.67) n=15
			Control	45.13 (3.64) n=15
				
		Posttest	CBT	55.31 (4.73) n=13
			SOC	53.00 (5.18) n=12
			Control	48.50 (4.62) n=14
				
		Follow up	CBT	56.76 (5.16) n=13
			SOC	62.75 (5.13) n=12
			Control	49.25 (5.11) n=12
				
Social Situations		Pretest	CBT	
				13.33 (8.16) n=15
			SOC	13.26 (1.75) n=15
			Control	13.20 (1.86) n=15
				
		Posttest	CBT	16.77 (1.36) n=13
			SOC	15.33 (1.49) n=12
			Control	14.50 (1.09) n=14

To compare the relative effectiveness of CBT versus SOC, post hoc tests revealed a significant difference between the effectiveness of CBT and SOC on general self-efficacy at both posttest and follow up ( D =-6.96 ; df =2, 43 ; p<0.01; D =-10,98 ; df = 2, 43; p<0.01 respectively). This means that SOC has been significantly more effective than CBT as far as general self-efficacy is concerned.

On the other hand, when analyzing results for situational self-efficacy, it was found that CBT was more effective than SOC on all three subscales of the ASES at posttest but not at follow up. The trend was in fact the opposite when comparing posttest with follow up in that SOC was found to be more effective on all three measures (Table 4).

**Table 3:** Comparative data of situational self-efficacy between two experimental groups with control group in posttest and follow up.

**Dependent variable**	**(I)** ** group**	**(J)** ** group**	**difference** **(I-J)**	**Error** **deviance**	**Sig.**
Emotional situations self-efficacy in post test	Control	CBT	-06.962^*^	1.510	0.001
		SOC	-03.308^*^	1.562	0.042
					
Emotional situations self-efficacy in follow up	Control	CBT	-04.794	2.647	0.008
		SOC	-10.989^*^	2.092	0.001
					
Social situations self-efficacy in post test	Control	CBT	-02.017^*^	0.381	0.01
		SOC	-00.897^*^	0.394	0.03
					
Social situations self-efficacy in follow up	Control	CBT	-03.084^*^	0.601	0.001
		SOC	-03.892^*^	0.475	0.001
					
grief situations self-efficacy in post test	Control	CBT	-02.520^*^	0.630	0.001
		SOC	-01.199	0.652	0.05
					
grief situations self-efficacy in follow up	Control	CBT	-01.047	0.589	0.05
		SOC	-02.467^*^	0.466	0.001

## Discussion

Results of the present study shows that both cognitive-behavioral and Trans theoretical Model were effective in that both significantly improved general and situational self-efficacy as compared with the control group. Also significant differences between the two therapeutic models were found. It can be concluded that that CBT seems to be significantly more effective than SOC in the short term but the opposite effect can be observed when self-efficacy is measured after a two month follow up. Hence SOC may have more permanent and long lasting effect on self-efficacy than CBT. 

**Table 4:** Comparative data's of situational self-efficacy between two experimental in posttest and follow up.

**Dependent variable**	**(I) group**	**(J) group**	**difference** ** (I-J)**	**Error deviance**	**Sig.**
Emotional self-efficacy in post test					
	CBT	SOC	-3.654^*^	1.652	0.034
					
Emotional self-efficacy in follow up					
	CBT	SOC	-6.195^*^	1.932	0.003
					
Emotional self-efficacy in post test					
	CBT	SOC	-1.120^*^	0.417	0.01
					
Emotional self-efficacy in follow up					
	CBT	SOC	-0.808	0.439	0.05
					
Emotional self-efficacy in post test					
	CBT	SOC	-0.690	0.04	1.321
					
Emotional self-efficacy in follow up					
	CBT	SOC	-1.420^*^	0.430	0.002
					

Results of the present study reiterate the findings reported by several authors (3,28,29) that CBT is an effective intervention technique for improving general self-efficacy in substance dependent patients (3). It can be argued that learning new and suitable behaviors for coping with addiction can create the ability and a sense of control in patients (3). CBT, thus providing new cognitive – behavioral coping skills the acquisition and use of which may lead to success in overcoming internal and external stresses and hence the amplitude of such successful experiences may help the formation of self-efficacy beliefs. Following this argument, it has been suggested by several authors that CBT and coping skills training method can be used successfully to improve general self-efficacy in addicted persons (30).

It was also found in the present study that CBT was effective for adolescents' self-efficacy improvement in high risk situations for substance use. This finding is similar to various research reports (31,33). It is generally believed that a sense of self-efficacy in high risk situations for substance use is considered to be an important factor in predicting coping quality with substance use (34). Therefore, training cognitive-behavior coping strategies improves situational self-efficacy and may be a stronger predictor of the success of the treatment than general self-efficacy. 

Stages of Change Model intervention were found to be effective on general self-efficacy in adolescents. Our finding is in line with several studies investigating the application of SOC to various unhealthy behaviors such as alcohol abuse (35,36). Apart from the present study, to date, the use of SOC in improving self-efficacy in adolescents has not been investigated. The present study supports the notion that SOC can be a suitable model for improving general self-efficacy. Interestingly, the present study found the group receiving SOC showed more improvement in general self-efficacy than in situational self-efficacy. That is to say, SOC treatment for general self-efficacy was more effective than self-efficacy in emotional and social high risk situations as measured in posttest and follow up.

In explaining this finding it can be pointed out that since the model promotes change over time, drawing from a variety of theoretical approaches and techniques to introduce change in behavior, its influence on the mental functioning of patients may spread and would not be limited to special problems in particular situations. In order to initiate effective and useful motivational, emotional and cognitive- behavioral changes, it is suggested that SOC, which is an amalgam of various psychotherapeutic approaches, may be a suitable model to be used by practitioners. 

Another interesting finding of the present study was the limited effectiveness of SOC in short term and its more long term influence on situational self-efficacy. It seems that SOC is by no means an intensive treatment technique. Hence, compared to other therapeutic methods, it does not seem to be as effective as may be expected in specific problem situations such as self-efficacy in emotional or social situations. However, it is capable of making more long term changes possible as far as coping with high risk situations is concerned. 

The results of the present study also support some findings which purport the possible long term effects of SOC with adolescent addicts, albeit indirectly (13). 

In one study, (30) self-efficacy was used as a predictor of treatment outcome in adolescent substance use disorders. The researchers asked whether perceived situational self-efficacy was itself differentially affected by the type of therapy. Subjects were assigned to either CBT condition, which focused on the enhancement of self-efficacy, or a non-CBT condition such as psycho-education (PET). Results showed that subjects showed significantly more self-efficacy in the CBT group. According to learning theory, it has been assumed that the mechanism of action responsible for the success of CBT relapse prevention is the acquisition and application of coping skills. Therefore, a pivotal objective of approaches based on social learning theory to the treatment of substance use disorders is to focus on the improvement of these deficits. Nevertheless, Joseph et al.’s hypothesis that subjects receiving CBT would show higher levels of situational self-efficacy in comparison to those receiving PET was not confirmed (31). It has been pointed out that the mechanism underlying the relationship between self-efficacy and better outcomes is still unclear (10) and that finding provide only ambivalent support for a social learning theory approach. Perhaps a combination of other factors such as readiness to change, expectancy, therapeutic alliance or engagement in treatment, are responsible for change in self-efficacy (32).

In a review of the literature (37) have reiterated the importance of self-efficacy in recovery from addiction: "perhaps the best effective treatments for addiction recovery are those that improve self-efficacy". It may be argued that since the Stages of change model believes in the process of change over time, such a focus on individual roles and responsibilities in resolving doubts and ambivalence during the change process and improving self-efficacy can make permanent changes through improving self-efficacy by means of change mechanisms during the change stages. On other hand, to explain the poor results of CBT treatment in follow up assessment, we can reiterate the assertion that behavioral changes do not necessarily increase a sense of self-efficacy (3). Of course, a sense of self-efficacy is formed by various processes over time.

CBT may be considered a short term and intensive treatment for specific problem areas in addicted patients. On the other hand, CBT is helpful in identifying, avoiding and coping with problems. It is useful in identifying and avoiding more risky situations which are difficult to cope with (15). Since the role of the patient is often ignored and the method used is deductive, the influence of interventions after the end of treatment may sometimes be weak. In short, findings of this research show that both therapeutic interventions have been effective on improving both general and situational abstinence self-efficacy. But an important finding of this research was the effectiveness of SOC on both general and situational self-efficacy at two month follow up. In addition, comparative analysis between two models' effects on self-efficacy dimensions showed that SOC was more effective than CBT on general self-efficacy in both short and long terms. While in the short term, CBT was more effective on situational abstinence self-efficacy than SOC, the effects of SOC on situational self-efficacy were more remarkable and permanent than CBT approach in long term follow up. 

 We would like to reiterate the point made by (38) that SOC focuses on change made by choice rather than the changes which may be initiated and maintained via conditioning techniques and methods. Hence, SOC relies on the individuals taking responsibility for their actions and choices in the therapeutic process and therefore, future research should consider testing and using this model as a central feature in the treatment of substance dependence and abuse. 

## Authors’ contributions

MJ conceived and designed the study as part of his Master’s thesis under the supervision of SS. SS prepared the English version of the document. Data was analyzed and the experiment was carried out by MJ and SS. AA reviewed the manuscript and helped in the analysis of the data. All authors read and approved the final manuscript.

## Notes

**Declaration of Interest: **None.

**Registration ID in IRCT: **IRCT201112138394N1  
